# Knowing when to stop: Aberrant precision and evidence accumulation in schizophrenia

**DOI:** 10.1016/j.schres.2017.12.018

**Published:** 2018-07

**Authors:** Roberto Limongi, Bartosz Bohaterewicz, Magdalena Nowicka, Aleksandra Plewka, Karl J. Friston

**Affiliations:** a Universidad Tecnológica de Chile INACAP, Chile; b Pontificia Universidad Católica de Valparaíso, Chile; c University of Social Sciences and Humanities, Department of Psychology of Individual Differences, Warsaw, Poland; d University of Social Sciences and Humanities, Warsaw, Poland; e Wellcome Trust Centre for Neuroimaging, Institute of Neurology, University College London, UK; f Department of Cognitive Neuroscience and Neuroergonomics, Institute of Applied Psychology, Jagiellonian University, Krakow, Poland

**Keywords:** Temporal prediction, Active inference, Response inhibition, Dysconnection hypothesis, Schizophrenia, Diffusion models

## Abstract

Predictive coding and active inference formulations of the dysconnection hypothesis suggest that subjects with schizophrenia (SZ) hold unduly precise prior beliefs to compensate for a failure of sensory attenuation. This implies that SZ subjects should both initiate responses prematurely during evidence-accumulation tasks and fail to inhibit their responses at long stop-signal delays. SZ and healthy control subjects were asked to report the timing of billiards-ball collisions and were occasionally required to withhold their responses. SZ subjects showed larger temporal estimation errors, which were associated with premature responses and decreased response inhibition. To account for these effects, we used hierarchical (Bayesian) drift-diffusion models (HDDM) and model selection procedures to adjudicate among four hypotheses. HDDM revealed that the precision of prior beliefs (i.e., starting point) rather than increased sensory precision (i.e., drift rate) drove premature responses and impaired response inhibition in patients with SZ. From the perspective of active inference, we suggest that premature predictions in SZ are responses that, heuristically, are traded off against accuracy to ensure action execution. On the basis of previous work, we suggest that the right insular cortex might mediate this trade-off.

## 1 Introduction

This paper evaluates a prediction of the dysconnection hypothesis (Friston, 1998, Friston et al., 2016) about aberrant sensory precision and compensatory effects on the precision of prior beliefs. We pursue this using the temporal estimation of unfolding visual events. The dysconnection hypothesis suggests that the psychopathology of schizophrenia (SZ) is mediated neurophysiologically by deficient modulations of synaptic gain or excitation-inhibition balance, thought to be caused by abnormal NMDA and dopaminergic neurotransmission (Laruelle et al., 2003). From a neurocomputational perspective, the hypothesis calls on the theoretical tenets of predictive coding (Friston and Kiebel, 2009, Rao and Ballard, 1999) and active inference (Friston et al., 2011).

Predictive coding equips the dysconnection hypothesis with a functional link between sensory precision and synaptic gain. Briefly, in predictive coding, the brain generates predictions at various levels in the cortical hierarchy. Higher levels send predictions to lower levels, which then reciprocate prediction errors (PEs) to higher levels, minimizing PEs and optimizing the ensuing predictions. Crucially, it is thought that the brain weighs PEs based on their reliability, or precision, which is thought to be reflected in the synaptic gain of neuronal populations reporting PEs (Friston, 2008). Put simply, a large synaptic gain represents precise ascending PEs, and vice versa.

Crucially, for PEs to optimize predictions effectively, they must be afforded by the appropriate precision; i.e., assigned the right degree of confidence. This is particularly important in hierarchical inference, where the precision of PEs at each level of the hierarchy determines the balance between prior beliefs and sensory evidence during evidence accumulation. An imbalance between sensory and prior precision can, in principle, lead to false perceptual (e.g., hallucinations) and conceptual inference (e.g., delusions), see also Moritz et al. (2015). The synaptic implementation of precision or synaptic gain control is therefore crucial for a veridical grip on the world, where it forms the computational homologue of attention (Feldman and Friston, 2010). The control of sensory precision is also particularly important for action.

In active inference, actions are prescribed by descending proprioceptive predictions that engage classical reflex arcs. These descending predictions provide the equilibrium or set points for motor reflexes that realize the intended or predicted movement (Adams et al., 2013a). However, this requires the attenuation of sensory (exteroceptive) PEs that would otherwise allow ascending (proprioceptive) PEs to revise predictions about the impending action. This attenuation is thought to be the computational homologue of sensory attenuation (Brown et al., 2013). In this sense, sensory attenuation is necessary for action. This follows from the fact that action is driven by descending predictions of what “I would sense if I made this movement”. A failure to attenuate proprioception would therefore preclude movement because prevents a suspension of attention to sensory evidence that “I am not moving”. Simulations of a failure to attenuate sensory precision produce bradykinesia and psychomotor poverty (Brown et al., 2013) and provide a straightforward explanation for empirical phenomena in conditions like Parkinson's disease and SZ (Adams et al., 2012, Adams et al., 2013b, Hughes et al., 2013, Oestreich et al., 2015).

A failure to attenuate sensory precision and a compensatory increase in prior precision has been proposed to explain hallucinations and delusions respectively (Adams et al., 2012, Bastos-Leite et al., 2015, Brown et al., 2013, Fogelson et al., 2014, Friston et al., 2016, Powers et al., 2017). The basic idea is that people with SZ are unable to attenuate the precision of sensory PEs; thereby exposing themselves to sensory evidence that cannot be ignored. This aberrant precision then induces a compensatory increase in the precision of PEs that underwrites prior beliefs at higher levels of the perceptual hierarchy. This aberrant precision formulation accounts for two fundamentally different sorts of false inference in SZ that can be thought of in terms of false negatives and false positives. A failure to attenuate sensory precision leads to negative symptoms and soft neurological signs in SZ (e.g., psychomotor poverty, resistance to illusions, failures of slow pursuit, attenuating mismatch negativity responses, etc., Adams et al., 2013b) that can be understood as a failure to elicit predictions (of sensations or movements) that are informed by prior beliefs. On the other hand, a compensatory increase in prior precision is thought to lead to positive symptoms (e.g., hallucinations and delusions, Powers et al., 2017) that represent prior beliefs that are afforded too much confidence. In short, the precision of sensory PEs, relative to prior beliefs, furnishes a theoretical framework for explaining negative and positive symptoms in SZ and testing predictions about accompanying cognitive and behavioral sequelae. Crucially, this framework can be related gracefully to evidence-accumulation schemes through precision. As we will see below, sensory precision controls the sensitivity to sensory evidence and therefore the rate at which it is accumulated (FitzGerald et al., 2015a, FitzGerald et al., 2015b).

Simulating, measuring, and modeling oculomotor behavior when SZ subjects track a moving object, suggests that aberrant sensory precision precludes the acquisition of prior beliefs based upon regular motion patterns (Adams et al., 2015, Adams et al., 2016, Adams et al., 2012). These prior beliefs normally allow people to predict *when* a moving object will reach a target (i.e., temporal estimation) (Barnes and Donelan, 1999, Fukushima et al., 2013, Heinen et al., 2005, Missal and Heinen, 2017). Interestingly, impaired temporal estimation is characteristic of subjects with SZ (Alústiza et al., 2017).

**Fig. 1:**
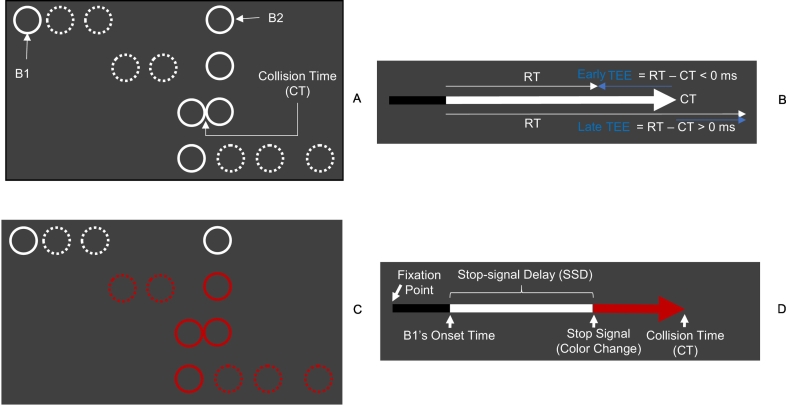
Response inhibition in TTC estimations. Go trial (A) and its timeline (B). Stop trial (C) and its timeline (D).

A task in which the aberrant encoding of sensory precision could affect this sort of temporal inference is the “time to collision” (TTC) task (i.e., estimating the time of the collision of a moving object with a stationary object, Fig. 1). Healthy subjects predict a TTC that is too early (indexed by a short response time, RT) when they can no longer track the motion of the moving object — and are therefore unable to update their beliefs about its trajectory. In this situation, their estimates are based largely on their prior experience. For example, their experience of responding prematurely leads them to believe that a loss will occur (in this sort of experimental setting, prior beliefs are usually induced by task instructions). This results in a large temporal estimation error (TEE); namely, RT minus collision time (Limongi and Pérez, 2017). It follows, that if aberrant precision control in SZ leads to a compensatory increase in the precision of prior beliefs, we should find a similar effect (i.e., large absolute TEEs or short RTs), even when visual motion information is available.

Predicting the TTC requires not only tracking the motion of the moving object but also preparing a response, putting it “on hold”, and “releasing” it a few milliseconds before the collision (to compensate for sensorimotor delays). Placing prepared actions “on hold”, requires response inhibition (Los, 2013). Interestingly, response inhibition is impaired in SZ (Hughes et al., 2012, Thakkar et al., 2011, Zandbelt et al., 2011). It is therefore possible that premature responses in SZ are associated with impaired response inhibition — and that this is associated with aberrant precision control.

Response inhibition is described phenomenologically by the (independent) horse-race (IHR) model (Logan and Cowan, 1984, Logan et al., 2014). In this model, two processes (stop and go) independently run towards a decision threshold. Each process comprises a RT (the RT_go_ and the stop-signal RT, SSRT), and the first process reaching the threshold wins the race. The delay between the go and stop signals (the stop-signal delay, SSD) affects the conclusion of the stop process relative to the conclusion of the go process. When RT_go_ > SSRT + SSD the agent inhibits the ongoing response whereas when RT_go_ < SSRT + SSD the agent fails to inhibit the response. The model assumes that go and stop processes are independent in terms of context (the same distribution of RT_go_ and RT_signal-respond_) and stochasticity (the across-trial variabilities of RT_go_ and SSRT are independent).

Based on the assumptions of the IHR model, subjects that respond quickly to a go signal are less likely to stop responses on stop trials with a long SSD. From the perspective of the “act-of-control” theory (Logan and Cowan, 1984, Logan et al., 2014), healthy subjects engage in “strategic slowing” to inhibit responses at long SSDs. On this view, SZ subjects prematurely release responses because they are unable to inhibit responses strategically. Alternatively, from the perspective of aberrant precision control, if overconfidence in prior beliefs causes short RTs (i.e., premature responses) and short RTs are associated with a failure to inhibit responses at long SSDs, then overconfidence in prior beliefs would necessarily impair response inhibition.

In the current study, we provide behavioral evidence that disambiguates these alternative (strategic slowing and aberrant precision) explanations for response deficits in SZ. Using model comparison, we show that premature responses are caused by elevated confidence in prior beliefs. Furthermore, to achieve our goal, we capitalized on the formal relationship between evidence-accumulation models of decision making, predictive coding (Bitzer et al., 2014, FitzGerald et al., 2015a, Hesselmann et al., 2010), and the IHR model of response inhibition (Logan et al., 2014, Verbruggen and Logan, 2009).

Evidence-accumulation models assume that subjects report a decision after accumulating evidence (Forstmann et al., 2016). In predictive coding, evidence accumulation corresponds to assimilating PEs; i.e., the accumulation of presynaptic afferent activity from neuronal populations encoding PEs (e.g., superficial pyramidal cells). Subjects with SZ would accumulate these inputs at a higher rate, which is the drift rate (v). Put simply, it represents the quality of the sensory evidence that – in the context of oculomotor pursuit – corresponds to the precision of sensory PEs in low levels of the cortical hierarchy (i.e., visual area 1, V1, Adams et al., 2016). A decision is made when the accumulated evidence reaches a threshold (a). Crucially, in predictive coding the starting point of the accumulation process (z) represents the subjective bias or confidence placed in prior beliefs (i.e., prior precision). We therefore hypothesized that subjects with SZ would show a greater subjective bias (i.e., increased prior precision) that would be accompanied by an increased drift rate (i.e., a failure to attenuate sensory precision). Conversely, the act-of-control theory predicts that impaired “strategic slowing” in SZ would be associated with a decreased decision threshold, accounting for short RTs (i.e., premature responses) and impaired response inhibition. In summary, we used phenomenological models of evidence accumulation to test the aberrant precision hypothesis in SZ, in the setting of a TTC task combined with a stop-signal task (Logan and Cowan, 1984, Logan et al., 2014).

## 2 Experimental methods

### 2.1 Participants

We used a between-groups design. The control group comprised fifteen healthy subjects (6 women, *M* age = 41, *SD* = 6.54), whereas the SZ group comprised fifteen patients with SZ (7 women, *M* age = 41.3, *SD* = 8.44). All subjects provided informed consent forms. The study was conducted according to the standards of the University of Social Sciences and Humanities and approved by the Institutional Ethics Committee. Subjects completed the Hospital Anxiety and Depression Scale and the Baratt Impulsiveness Scale (BIS-11). Mini-Mental State Examination, and structured interview (based on ICD-10 and DSM-5 criteria of substance dependence) were used to asses cognitive functioning and history of substance abuse. Control subjects denied a history of drug usage, neurological, or somatic disorders. SZ subjects did not use non-alcoholic psychoactive substances during the 12 months before the study, were taking atypical antipsychotics drugs (e.g., olanzapine), and did not have pronounced positive symptoms or psychomotor agitation. The SZ group had a higher mean BIS score (*M* = 66.53, *SD* = 4.07) than the control group (*M* = 50.60, *SD* = 8.52), *t*_(20.07)_ = − 6.53, *p* < 0.001. Moreover, compared with the control group the SZ group exhibited higher levels of anxiety (*M* = 12.07, *SD* = 2.19 vs *M* = 4.07, *SD* = 3.17; *t*_(28)_ = − 8.04, *p* < 0.001) and depression (*M* = 10.20, *SD* = 2.48 vs *M* = 4; *SD* = 3.09; *t*_(28)_ = − 6.05, *p* < 0.001).

### 2.2 Task, stimuli, and conditions

On each trial, a fixation point appeared at the center of a computer screen for 150, 200, 250, 300, or 350 ms (randomly varied across trials). Then, two white circles (B1 and B2, 1.34 cm in diameter) appeared on the left (B1) and center (B2) of the screen and remained stationary for 500 ms. Following the appearance of the circles, B1 moved towards B2 at a constant speed (17.32 deg/s) until reaching the left-most edge of B2 (1000 ms later). At this point, B1 stopped and B2 moved in the same direction and with the same speed as B1. B2 stopped after reaching the right-most side of the screen. Both circles remained visible until 2700 ms had elapsed, from the onset of the animation. This sequence of stimuli creates the visual impression of one billiard ball colliding with another and imparting its momentum (Fig. 1).

Subjects performed go and stop trials. On go trials, they pressed the space bar when they judged B1 would collide with B2. On stop trials, the color of B1 and B2 changed from white to red, cuing subjects to withhold their responses. The red stop signal was presented with a delay (the SSD) after the onset of B1 motion. The SSD changed across trials, increasing or decreasing 30 ms after successful (signal stop) and unsuccessful (signal respond) trials respectively. With this (staircase) procedure, subjects reached an SSD for which they responded at 50% to a stop signal (SSD_50_). Subjects performed one familiarization block and five experimental blocks (30 go and 10 stop trials per block).

### 2.3 Data analysis

We performed three sets of analysis. First, we evaluated between-groups differences in temporal prediction accuracy indexed by the absolute TEE. We fit a mixed-effects linear model to all go-trial data points. The model included one regressor encoding the group effect (SZ and control), one regressor encoding the sign of the response (i.e., early or late; early responses were defined as TEE ≤ 0 ms), and one regressor encoding the Group × Sign interaction. Subjects were included as random effects.

Second, we also used mixed-effects models to evaluate between-groups differences in the SSD_50_ and in the SSRT by regressing subjects' SSD_50_ means and SSRTs on group (as a fixed effect) and subjects (as random effects). Subjects' SSRTs were computed via the mean method whose reliability (Verbruggen et al., 2013) was assessed by both regressing RT_go_ on trial number (as a fixed effect) and subjects (as random effects) and quantifying the skewness of the RT_go_ distributions. We also evaluated the context-independence assumption of the IHR model, by testing for differences between RT_signal-respond_ and RT_go_ (RT_signal__-__respond_ < RT_go_).

Finally, we fit four hierarchical drift-diffusion models (HDDM, Wiecki et al., 2013) of one-choice RT (Ratcliff and Van Dongen, 2011) to adjudicate between four competing hypotheses about the between-groups difference in RT_go_. Model 1 represented our hypothesis that “z” (the starting point or the precision of prior beliefs) and “v” (the drift rate or the precision of sensory PEs) varied across groups. We used a series of reduced models (models 2 and 3) to ensure that simpler models did not provide a better account of the data – that would otherwise be over fitted by the full model. In model 2, “z” varied across groups whereas “v” was fixed, meaning that the difference in RT_go_ could be caused only by aberrant prior beliefs. Conversely, in model 3 “v” varied across groups whereas “z” was fixed, meaning that the difference in RT_go_ could be caused only by aberrant sensory precision estimation. Finally, model 4 represented the hypothesis that difference in RT_go_ would be reflected in the decision threshold “a” — representing the effect of strategic adjustments or strategic slowing (Verbruggen and Logan, 2009) predicted by the act-of-control theory (Logan et al., 2014). Therefore, this parameter varied across groups whereas “z” and “v” were fixed. All four models included “t” (the non-decision time) as a fixed parameter.

To avoid negative drift rates, we did not estimate the inter-trial variability of “v” and “t” (Ratcliff and Van Dongen, 2011). Furthermore, we did not estimate inter-trial variability of “z”, because it does not improve model performance (Ratcliff and Van Dongen, 2011). Each model was fitted by drawing 10,000 samples using Markov chain Monte Carlo estimation of the posterior distribution over model parameters (discarding the first 200 samples). To choose the winning model, we used the deviance information criterion (DIC) as an approximation to Bayesian model evidence. We report parameter estimates of the winning model and the posterior proportion (PP) in which the free model parameter was larger in the SZ group.

## 3 Results

**Fig. 2:**
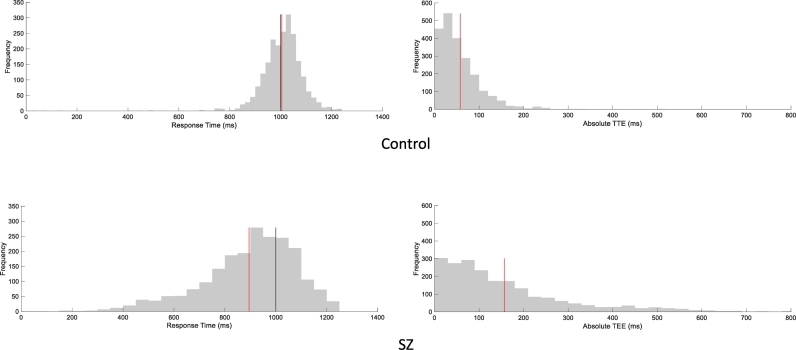
Distributions of RT_go_ and absolute TEEs. RT_go_ distributions are left skewed (skewness__SZ_ = − 0.87, skewness__control_ = − 2.74) Collision time (black vertical lines), mean values (red vertical lines).

Go trials with RT > 1250 ms (2.7%) and go trials with RT < 0 ms (1.6%) were excluded. Table 1 shows the relevant summary statistics. The SZ group made larger absolute TEEs (*β* = 42.15, *SE* = 9.67, *t*_(27.84)_ = 4.36, *p* < 0.0001, 95% CI = [22.34, 61.96]). Both groups made larger absolute TEEs in early predictions than in late predictions (*β* = 10.18, *SE* = 1.75, *t*_(4291)_ = 5.83, *p* < 0.0001, 95% CI = [6.76, 13.60]). However, early predictions were more inaccurate in the SZ group (*β* = 9.87, *SE* = 1.75, *t*_(4291)_ = 5.65, *p* < 0.0001, 95% CI = [6.45, 13.13], Fig. 2).

**Table 1:** Relevant summary statistics.

	Mean (standard deviation)	
	SZ	Control
Abs TEE ms	151.61 (139.84)	60.82 (61.47)
RT_go_ ms	898.84 (134.31)	1004.51 (29.93)
RT_Signal-Respond_ ms	856.44 (138.82)	980.36 (48.51)
Inhibition accuracy %	48 (50)	51 (50)
SSD_50_ ms	590.14 (174.74)	728.67 (66.60)
SSRT ms	308.7 (76.38)	275.84 (55.26)
z	0.43 (0.12)	0.30 (0.12)
a	8.86 (5.16)	
t	0.28 (0.91)	
v	8.48 (0.2)	

The SZ group responded faster than the control group on go trials (*β* = 52.86, *SE* = 17.82, *t*_(27.98)_ = 2.97, *p* = 0.006, 95% CI = [16.35, 89.36]). Both groups responded faster on signal-respond trials than on go trials (*t*_(14)control_ = 1.91, *p* = 0.038, *t*_(14)SZ_ = 2.82, *p* = 0.01). Neither between-groups difference in SSRT (*t*_(28)_ = 1.35, *p* = 0.19) nor effect of trial on RT_go_ (*β*_SZ_ = − 0.20, SE = 0.13, *t*_(13.94)_ = 1.51, *p* = 0.15, 95%, CI = [− 0.49, 0.08]; *β*_control_ = 0.008, SE = 0.06, *t*_(13.87)_ = 0.12, *p* = 0.9, 95%, CI = [− 0.13, 0.14]) was detected. Both groups reached approximately 50% of response inhibition, and the SSD_50_ was smaller in the SZ group (*β* = − 69.26, SE = 24.14, *t*_(28)_ = 2.87, *p* = 0.007, 95%, CI = [19.81, 118,72]).

Finally, DIC-based model comparison selected model 2 as the winning HDDM (DIC_model-1_ = − 6629, DIC_model-2_ = − 6785, DIC_model-3_ = − 6579, DIC_model-4_ = − 6629). The “z” parameter (starting point) was larger in the SZ group (PP_z_SZ > *z*_Control_ = 0.999). In summary, the best explanation for deficits in response inhibition was differences in the starting points of evidence accumulation that speak to an aberrant precision or confidence in prior beliefs.

## 4 Discussion

**Fig. 3:**
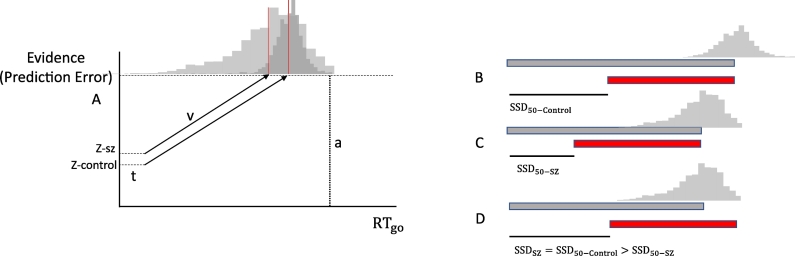
Drift-diffusion (A) and horse-race (B-D) representations of go responses. (A) Both groups showed the same decision threshold (a) and the same drift rate (v). But, higher precision of prior beliefs (z) caused the SZ group to accumulate less evidence. Therefore, the SZ group responded prematurely. The control group delayed responses, which was reflected in longer RT_go_ (gray horizontal bar) and longer SSD_50_ (thin black horizontal bar, B). The SZ group responded prematurely (shorter RT_go_ and SSD_50,_ C). Therefore, they had difficulty withholding prepared responses (i.e., predictions) at longer SSDs (e.g., the SSD_50_ of the control group) because the finishing time of the stop process was delayed (red horizontal bar)— relative to the finishing time of the go process (D). SSD_50-Control_ (SSD_50_ of the control group), SSD_50-SZ_ (SSD_50_ of the SZ group). Drift-diffusion (A) and horse-race (B-D).

The decision threshold and the evidence-accumulation rate were the same in SZ and control subjects (Fig. 3A). However, increased confidence in prior beliefs drove SZ subjects to respond prematurely, causing large absolute TEEs and a reduced probability of withholding actions on stop trials at long SSD (Fig. 3B–D). These results are congruent with studies suggesting that SZ patients “jump to conclusions” by gathering an inappropriate amount of information — and that they are overconfident about their conclusions (Evans et al., 2015). Furthermore, the results show for the first time a relationship between impaired temporal prediction and impaired response inhibition in SZ that is underwritten by an increased confidence in prior beliefs rather than by a decreased in the decision threshold (as predicted by the act-of-control theory).

Some studies using the stop-signal task report slow SSRT in SZ (Hughes et al., 2012, Thakkar et al., 2011). However, in accord with our results, one study reports no SSRT difference between SZ and healthy controls (Zandbelt et al., 2011). These disparate results might arise from different SSRT estimation methods: the mean method is thought to be unreliable when either the RT_go_ distribution is skewed to the right or when RT_go_ increases across trials (Verbruggen et al., 2013). These two conditions were not detected in our data. However, we note that although the IHR model does not assume any specific RT_go_ distribution, the reliability of the mean method has not been evaluated when the distribution is left skewed (Fig. 2). Therefore, future studies using the current task should evaluate whether other SSRT estimation methods (e.g., the integration method) yield no between-groups difference in SSRT.

SSRT might also differ across tasks demands (Logan et al., 2014). The stop-signal task requires subjects to respond as quickly as possible after the go-signal onset time. In the current task, B1's onset played the role of the go signal — to compute RT_go_ objectively. However, subjects triggered the go process based upon temporal predictions of an impending stimulus (B2's onset). Therefore, they placed responses (i.e., predictions) “on hold” until a critical time had elapsed. In the stop-signal task, predictions must be prevented — which is generally achieved by using a forced two-choice RT paradigm with variable foreperiods (Logan et al., 1984, Niemi and Näätänen, 1981). Therefore, it would be worth investigating whether a two-choice temporal prediction task with variable foreperiods (e.g., an action update task, Limongi et al., 2016) replicates the current results.

One could argue that proactive adjustments (Verbruggen and Logan, 2009) could account for responses with long RT_go_ in the control group, compared with the SZ group, especially because proactive inhibition is impaired in SZ (Zandbelt et al., 2011). However, such adjustments affect the right tail of the RT_go_ distribution. Our data neither showed such an effect nor supported the HDDM representing this hypothesis. Interestingly, previous evidence-accumulation models of proactive adjustments have not included a starting-point parameter. Therefore, our results challenge the act-of-control hypothesis that strategic slowing is associated with higher evidence-accumulation threshold.

From the perspective of an evidence-accumulation theory, a premature starting time of the go process relates to a decision bias. From the perspective of active-inference, this reflects an increase in the precision of prior beliefs, which allows subjects with SZ to execute responses. Heuristically, one could imagine that subjects with SZ trade temporal estimation accuracy to ensure action execution — following a failure to attenuate sensory precision. However, our HDDM results were a little surprising because we expected not only between-groups difference in (the precision of) prior beliefs but also in the drift rate (i.e. sensory precision). We did not find evidence for changes in drift rate. A possible explanation is that the drift rate represents the precision of evidence-accumulation rate at higher cortical levels whereas aberrant *sensory* precision is limited to lower levels (e.g., V1, Adams et al., 2016). This is an interesting hypothesis that we will address in future studies. However, the picture that this unexpected finding may speak to is that the decision of *when* to report the prediction is resolved at the same high level in the cortical hierarchy. This follows because both groups appear to have adopted the same decision threshold. As discussed below, we suggest that the right insular cortex might compute this decision threshold.

Relatively few studies have examined temporal prediction processes in SZ (Koreki et al., 2015) and such studies have focused on the auditory modality (Rentzsch et al., 2015). However, impaired timing in SZ does not appear to be domain specific (Ciullo et al., 2016). Furthermore, a recent meta-analysis (Alústiza et al., 2017) implicated increased activation in the right insular cortex of patients with SZ who showed impaired time processing. In concordance, Limongi et al. (2013) reported the association between large TEEs and right insular activity in healthy subjects. Because absolute TEE is a proxy of (inverse) precision (cf., sum of squared error, FitzGerald et al., 2015a, Limongi et al., 2015), these facts speak to the right insula as a perception-action integration hub that is crucial for valenced (homeostatic or allostatic) action (Pezzulo et al., 2015). This follows because the insula is in a position to integrate ascending interoceptive (Gu et al., 2013) and sensory (Limongi et al., 2016) PEs with descending prefrontal predictions (i.e., prior beliefs). See also Furl and Averbeck (2011) for a discussion of the role of the insula in evidence accumulation and then valanced decisions.

In summary, the empirical findings confirmed both predictions of a dysconnection hypothesis about aberrant precision estimation: overly precise prior beliefs cause both premature responses and impaired response inhibition. The increased precision at higher hierarchical levels may be reflected in higher postsynaptic gain and activations in the right insula — and an accompanying decreased sensitivity to ascending PEs. This formulation is consistent with weak connections between sensory areas and the insula (Limongi and Pérez, 2017, Limongi et al., 2016, Limongi et al., 2015).

In this work, we only measured manual responses and only used behavioral models. However, the aberrant precision hypothesis has been confirmed by oculomotor data and neural models. Therefore, we have assumed that oculomotor and manual behaviors do not differ in their underlying mechanisms. This assumption should be tested in future studies by combining eye tracking and dynamic causal models of eye responses (Adams et al., 2015), which would allow to quantify and test subjects' prior beliefs based on oculomotor behavior during an eye-hand task. A further limitation was that we only measured visual processing. However, impaired temporal perception in SZ appears to be a multisensory deficit (Stevenson et al., 2017). A future study should address this limitation by using a multisensory task.

## Role of the funding source

Roberto Limongi is funded by Universidad Tecnológica de Chile INACAP. Karl J. Friston is funded by a Wellcome Trust Principal Research Fellowship (Ref: 088130/Z/09/Z).

## Contributors

Roberto Limongi designed the study, analyzed the data, and wrote the first draft of the manuscript. Bartosz Bohaterewicz performed the study and contributed to the final manuscript. Magdalena Nowicka performed the study, supervised the study, and contributed to the final manuscript. Aleksandra Plewka performed the study and contributed to the final manuscript. Karl J. Friston contributed to the final manuscript. All authors approved the manuscript.

## Conflicts of interest

All authors declare no conflicts of interest.
